# Development of a prognostic model to identify the metastatic nasopharyngeal carcinoma patients who may benefit from chemotherapy combination PD-1 inhibitor

**DOI:** 10.3389/fimmu.2023.1069010

**Published:** 2023-01-17

**Authors:** Guo-Ying Liu, Nian Lu, Wei-Xin Bei, Wang-Zhong Li, Hu Liang, Wei-Xiong Xia, Yan-Qun Xiang, He-Rui Yao

**Affiliations:** ^1^ Guangdong Provincial Key Laboratory of Malignant Tumor Epigenetics and Gene Regulation, Medical Research Center, Sun Yat-Sen Memorial Hospital, Sun Yat-Sen University, Guangzhou, China; ^2^ Department of Radiotherapy, Sun Yat‐sen Memorial Hospital, Sun Yat‐sen University, Guangzhou, China; ^3^ Department of Nasopharyngeal Carcinoma, Sun Yat-Sen University Cancer Center, Guangzhou, Guangdong, China; ^4^ State Key Laboratory of Oncology in South China, Collaborative Innovation Center for Cancer Medicine, Guangdong Key Laboratory of Nasopharyngeal Carcinoma Diagnosis and Therapy, Sun Yat-Sen University Cancer Center, Guangzhou, China

**Keywords:** nasopharyngeal carcinoma, PD-1 inhibitor, prognostic model, propensity matching analysis, progression-free survival

## Abstract

**Background:**

We aimed to establish a prognostic model to identify suitable candidates for chemotherapy combination PD-1 inhibitor in metastatic nasopharyngeal carcinoma (NPC) patients.

**Patients and methods:**

In this retrospective study, we included 524 patients (192 patients treated with chemotherapy combination PD-1 inhibitor and 332 received chemotherapy alone as first-line regimen) with metastatic NPC between January 2015 and March 2021. We developed a prognostic model to predict progression-free survival (PFS). A model-based trees approach was applied to estimate stratified treatment effects using prognostic scores and two well-matched risk groups (low-risk and high-risk) were created using propensity score matching.

**Results:**

A prognostic nomogram was established with good accuracy for predicting PFS (c-index values of 0.71; 95% confidence interval, 0.66-0.73). The survival curves were significantly different between low-risk and high-risk groups (median PFS: 9.8 vs. 22.8 months, P < 0.001, respectively). After propensity matching analysis, chemotherapy combination PD-1 inhibitor was significantly associated with superior PFS as compared with chemotherapy alone (median PFS, 10.6 versus 9.3 months, P = 0.016) in the high-risk group. However, no significant difference between chemotherapy combination PD-1 inhibitor and chemotherapy was observed (P = 0.840) in the low-risk groups.

**Conclusions:**

Our novel prognostic model was able to stratify patients with metastatic NPC into low-risk or high-risk groups and identify candidates for PD-1 inhibitor therapy. These results are expected to be confirmed by a prospective clinical trial.

## Introduction

Nasopharyngeal carcinoma (NPC) is one of the most common head and neck cancer in southeast Asia, with an annual incidence of approximately 133,354 cases worldwide (1). With the advancement of radiotherapy and imaging technology, local control rate exceeds 90%, distant metastasis remains the leading cause of death in NPC (2, 3). At present patients with metastatic NPC often respond well to first-line chemotherapy (with an objective response of 45%-70%) (4, 5). However, the vast majority of the patients will progression soon after the first-line chemotherapy.

NPC is mainly associated with EB virus infection in endemic areas (6). Therefore, tumor regions have abundant lymphocyte infiltration and elevated programmed death-ligand 1 (PD-L1) expression, making immunotherapy a promising option for the treatment of NPC (7, 8). Recently, two double-blind, randomized phase III clinical trial demonstrated that PD-1 inhibitor added to chemotherapy for patients with recurrent or metastatic NPC provided superior progression-free survival (PFS) than chemotherapy alone (9, 10). Due to the heterogeneity between patients, the benefits of immunotherapy may be inconsistent. Given our lack of clear indications which patients with metastatic NPC might benefit from PD-1 inhibitor, it is necessary to be properly stratified beforehand to avoid unnecessary toxic effects from overtreatment. Furthermore, due to the high cost of cancer treatment, socioeconomic differences have been shown to influence treatment choices and survival outcomes for cancer patients (11). It is urgent to develop a clinically useful tool to predict PFS in heterogeneous patient populations and to aid treatment decisions.

The present study attempts to develop a prognostic tool to identify patients who could benefit more from chemotherapy combination PD-1 inhibitor and to make personalized predictions in metastatic NPC patients.

## Materials and methods

### Study population

We conducted this retrospective study of metastatic NPC patients treated with chemotherapy with or without PD-1 inhibitor as first-line regimen at the Sun Yat-sen University Cancer Center (SYSUCC) between January 2015 and March 2021. Patients who met the following inclusion criteria were enrolled:1) histologically confirmed NPC with metastatic disease; 2) platinum-based chemotherapy as first-line treatment with or without PD-1 inhibitor; 3) measurable metastatic lesions; 4) available baseline or post-chemotherapy radiologic evaluation data; 5) and no previous malignancies at other sites. The study received the ethics approval from the institutional review committee, and all participants written informed consent.

### Baseline evaluation and treatment

The detailed medical history, hematologic examination, biochemical analysis and Epstein-Barr Virus (EBV) DNA at baseline before chemotherapy (within 30 days before the treatment) were extracted from electronic medical records. All patients underwent a flexible nasopharyngoscopy; and magnetic resonance imaging (MRI) or computed tomography (CT) of the nasopharynx and neck for primary tumor staging. Distant metastasis staging was completed with CT/MRI examination of the chest and abdomen and skeletal scintigraphy. Positron emission tomography was recommended for patients if persistent distant metastasis was suspected.

All patients received platinum-based chemotherapy as first-line regimen. The main chemotherapy regimen in this study included platinum and 5-fluorouracil (PF); taxane and platinum (TP); taxane, platinum and 5-fluorouracil (TPF); gemcitabine and platinum (GP). **Supplementary Material 1** summarizes detailed information related to treatment.

### Outcomes and follow-up

Progression-free survival (PFS), defined as the time from the start date of treatment to disease progression or death from any cause, was the primary endpoint of the study. During follow-up, clinical examinations, fiberoptic nasopharyngoscopy, EBV DNA, MRI, or positron emission tomography CT were routinely performed. Follow-up was performed at least every 3 months after treatment completion. Patients who lost follow-up at last contact were censored.

### Statistical analysis

Differences between groups were analyzed using the Chi-square test or Fisher’s exact test for categorical variables. To reduce the effect of selection bias in treatment, propensity score matching (PSM) method was used to control for differences in baseline characteristics between groups. Kaplan-Meier method was used to compare PFS and Cox model to determine independent prognostic factors. A predictive model was developed by identifying independent risk factors and displaying them in a nomogram. The identification, predictive accuracy and calibration of the prognostic model were evaluated using a conformance index (c-index) and calibration maps. All statistical analyses were performed using R software package (version 4.5.0). A *P* value less than 0.05 was considered statistically significant.

## Results

### Patient characteristics and treatment outcomes

Of 524 patients with metastatic NPC who were treated first-line chemotherapy with PD-1 inhibitor (n = 192) or without PD-1 inhibitor (n = 332) were included. The patients included 420 men (80.2%) and 104 women (19.8%), with a median age of 46 years (interquartile range, 38-53 years). As is typical of endemic areas, 513 (97.8%) patients had World Health Organization (WHO) type III disease. Among all the patients, 241 (46.0%) patients had a primary disease, and 283 (54.0%) had recurrent/metastatic disease. **Table 1** shows the detailed clinicopathologic characteristics.

**Table 1:** Clinicopathologic characteristics and univariate analysis of PFS in the 524 patients.

Variables	No. of patients (%)	Univariate analysis	
		HR (95% CI)	*P*
Gender			
Male	420 (80.2)	Reference	
Female	104 (19.8)	0.971 (0.747 to 1.262)	0.827
Age			
<45 year	224 (42.7)	Reference	
≥45 year	300 (57.3)	1.205 (0.978 to 1.484)	0.080
ECOG PS			
0-1	496 (94.7)	Reference	
2	28(5.4)	1.236 (0.759 to 2.012)	0.394
Smoking status			
Yes	184 (35.1)	Reference	
No	340 (64.9)	0.901 (0.728 to 1.117)	0.901
Disease status			
Primary metastatic	241 (46.0)	Reference	
Recurrent metastatic	283 (54.0)	1.313 (1.068 to 1.614)	0.010
Number of metastatic sites			
Oligo	170 (32.4)	Reference	
Multiple	354 (67.6)	2.593 (2.029 to 3.314)	< 0.001
Number of metastatic organs			
Single	299 (57.1)	Reference	
Multiple	225 (42.9)	1.579 (1.268 to 1.939)	< 0.001
Liver metastatic			
Absent	350 (66.8)	Reference	
Present	174 (33.2)	1.504 (1.215 to 1.861)	< 0.001
Bone metastatic			
Absent	255 (48.7)	Reference	
Present	269 (51.3)	0.982 (0.800 to 1.206)	0.864
Lymph nodes metastatic			
Absent	388 (74.0)	Reference	
Present	136 (26.0)	1.414 (1.129 to 1.772)	0.003
Lung metastases			
Absent	316 (60.3)	Reference	
Present	208 (39.7)	1.247 (1.013 to 1.535)	0.037
Alkaline phosphatase (U/L)			
<110	437 (83.4)	Reference	
≥110	87 (16.6)	1.691 (1.306 to 2.190)	< 0.001
C-reactive protein (g/mL)			
<3	229 (43.7)	Reference	
≥3	295 (56.3)	1.277 (1.037 to 1.572)	0.021
EBV DNA (copies/mL)			
<1000	158 (30.2)	Reference	
≥1000	366 (69.8)	1.879 (1.479 to 2.389)	< 0.001
Lactate dehydrogenase (U/L)			
<245	393 (75.0)	Reference	
≥245	131 (25.0)	2.019 (1.611 to 2.531)	< 0.001

The values presented are median (interquartile range) or number (%). ECOG PS, Eastern Cooperative Oncology Group Performance Status. CI, confidence interval; HR, hazard ratio; EBV, Epstein–Barr virus.

As of May 2022, with a median patient follow-up time of 34.7 months (interquartile range, 23.8–58.3 months), 367 (70.0%) patients developed disease progression, and 160 (30.5%) patients had died. Overall, the median PFS of the entire cohort was 13.0 (95% CI 11.9-14.1) months, with 1-year, 2-year and 3-year PFS probability being 53.7%, 29.4% and 23.9%, respectively.

### Development of the prognostic model

**Figure 1:**
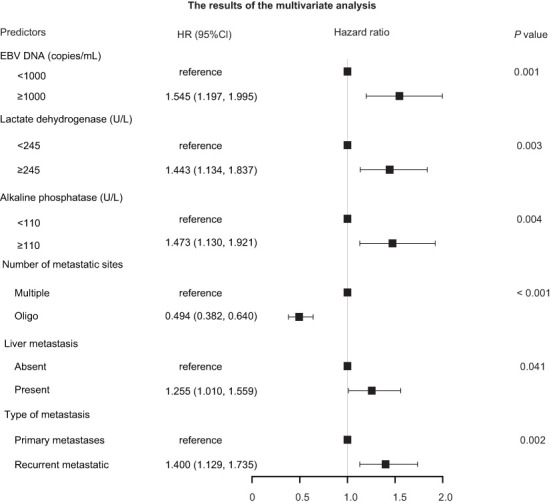
Forest plots depicting the multivariate association of clinicopathological characteristics with progression-free survival. CI, confidence interval; EBV, Epstein–Barr virus; HR, hazard ratio.

**Figure 2:**
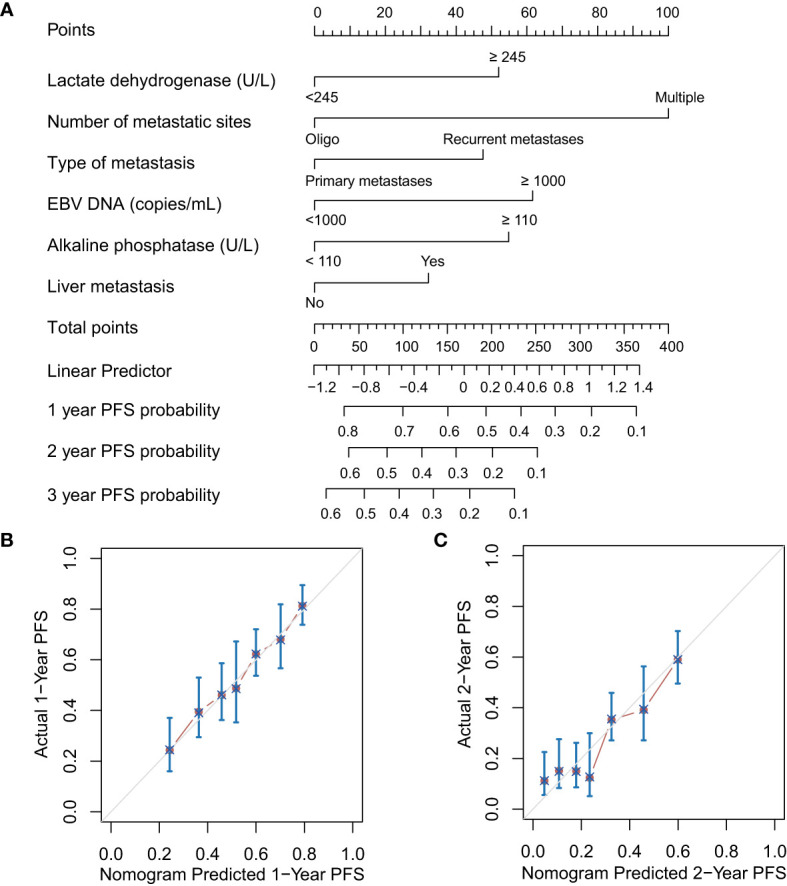
Prognostic nomograms **(A)** and calibration plots of survival probabilities at 1 years **(B)** and 2 years **(C)** in patients with metastatic NPC. EBV, Epstein–Barr virus; PFS, progression-free survival.

The results of univariate Cox regression models are summarized in **Table 1**. In multivariate analysis, high baseline alkaline phosphatase (HR1.473, 95%Cl 1.130-1.921, P = 0.004), lactate dehydrogenase (HR 1.443, 95%Cl 1.134-1.837, P = 0.003), EBV DNA (HR 1.545, 95%Cl 1.197-1.995, P = 0.001), present with liver metastatic (HR 1.255, 95% Cl 1.010-1.559, P = 0.041), number of metastatic sites (HR 0.494, 95%Cl 0.382-0.640, P < 0.001) and disease status (HR 1.400, 95%Cl 1.129-1.735, P = 0.002) were independent statistically significant prognostic factors for PFS (**Figure 1**). Next, we constructed a prediction model based on the above six independent prognostic factors and represented it with a nomogram. With c-index values of 0.71 (95% CI, 0.66-0.73), the prognostic nomogram showed good accuracy in predicting PFS, respectively. Moreover, calibration plots for the probabilities of 1- and 2-year PFS [**Figures 2B, C**] showed ideal agreement with the nomogram predictions for PFS [**Figure 2A**], respectively.

### Risk stratification

**Figure 3:**
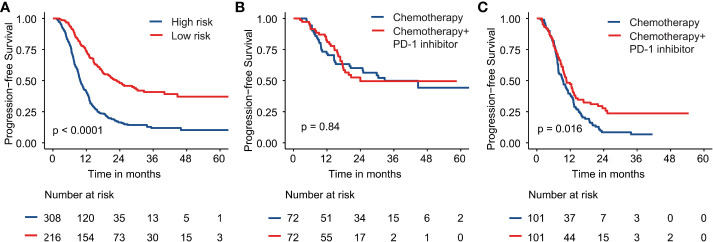
Kaplan–Meier survival curves are shown for progression-free survival for **(A)** high and low-risk groups; **(B)** the low-risk group with chemotherapy plus PD-1 inhibitor or chemotherapy alone; **(C)** the high-risk group with chemotherapy plus PD-1 inhibitor or chemotherapy alone.

Based on probability points of nomograms for disease progression, all patients were classified as two risk groups: low-risk group (216 patients) and high-risk group (308 patients). Compared with the low-risk group, the high-risk group had a greater number of metastatic sites (target and nontarget lesions), number of metastatic organs (violated organs), present liver metastatic, lymph nodes metastatic, elevated EBV DNA, C-reactive protein, alkaline phosphatase and lactate dehydrogenase levels (P < 0.001 for all). The baseline characteristics of high-risk and low-risk groups are shown in **Supplementary Table 1**. The patients in high-risk group were significantly associated with unfavorable prognostic factors. The median PFS of low-risk and high-risk patients was 22.8 (95% Cl 16.0-29.6) months and 9.8 (95% Cl 9.0-10.7) months, respectively (P < 0.001, **Figure 3A**).

### Benefits of adding ICI to chemotherapy in each risk group

All of the above validated predictors (alkaline phosphatase, disease status, liver metastatic, EBV DNA, number of metastatic sites and lactate dehydrogenase) based on the results of PFS in multivariate analysis were selected for inclusion in the propensity score analysis. Using the PSM method, two matched risk groups were created to compare PD-1 inhibitor plus chemotherapy with chemotherapy alone groups (all P > 0.05). **Table 2** lists detailed baseline characteristics for all risk groups. After PSM, chemotherapy combined PD-1 inhibitor revealed a significant improvement for PFS compared with chemotherapy alone in the patients with high-risk (median PFS, 10.6 versus 9.3 months, P = 0.016; **Figure 3C**). However, no significant difference was observed between chemotherapy combined PD-1 inhibitor and chemotherapy alone group in the patients with low-risk (P = 0.840; **Figure 3B**).

**Table 2:** The baseline characteristics of the patients treated with chemotherapy combination PD-1 inhibitor or chemotherapy alone in each risk group based on the propensity score method.

Characteristic	Low-risk group			High-risk group		
	Chemotherapy	Chemotherapy + PD-1 inhibitor	*P* value	Chemotherapy	Chemotherapy + PD-1 inhibitor	*P* value
Gender			0.548			0.285
Male	58 (80.6)	54 (75.0)		85 (84.2)	78 (77.2)	
Female	14 (19.4)	18 (25.0)		16 (15.8)	23 (22.8)	
Age			0.405			0.886
<46 year	32 (44.4)	38 (52.8)		42 (41.6)	40 (39.6)	
≥46 year	40 (55.6)	34 (47.2)		59 (58.4)	61 (60.4)	
ECOG PS			1.000			0.537
0-1	68 (94.4)	67 (93.1)		97 (96.0)	94 (93.1)	
2	4 (5.6)	5 (6.9)		4 (4.0)	7 (6.9)	
Smoking status			0.857			0.880
Yes	23 (31.9)	21 (29.2)		33 (32.7)	31 (30.7)	
No	49 (68.1)	51 (70.8)		68 (67.3)	70 (69.3)	
Type of metastasis			1.000			1.000
Primary metastases	30 (41.7)	29 (40.3)		42 (41.6)	42 (41.6)	
Recurrent metastases	42 (58.3)	43 (59.7)		59 (58.4)	59 (58.4)	
Number of metastatic sites			1.000			1.000
Oligo	55 (76.4)	56 (77.8)		0	0	
Multiple	17 (23.6)	16 (22.2)		101 (100.0)	101 (100.0)	
Number of metastatic organs			0.305			0.671
Single	60 (83.3)	54 (75.0)		47 (46.5)	43 (42.6)	
Multiple	12 (16.7)	18 (25.0)		54 (53.5)	58 (57.4)	
Liver metastasis			1.000			1.000
Absent	60 (83.3)	60 (83.3)		53 (52.5)	53 (52.5)	
Present	12 (16.7)	12 (16.7)		48 (47.5)	48 (47.5)	
Lung metastasis			1.000			0.468
Absent	43 (59.7)	42 (58.3)		66 (65.3)	60 (59.4)	
Present	29 (40.3)	30 (41.7)		35 (34.7)	41 (40.6)	
Bone metastasis			1.000			0.777
Absent	43 (59.7)	44 (61.1)		46 (45.5)	43 (42.6)	
Present	29 (40.3)	28 (38.9)		55 (54.5)	58 (57.4)	
Lymph nodes metastases			0.661			0.755
Absent	61 (84.7)	58 (80.6)		71 (70.3)	74 (73.3)	
Present	11 (15.3)	14 (19.4)		30 (29.7)	27 (26.7)	
Alkaline phosphatase (U/L)			1.000			1.000
<110	70 (97.2)	69 (95.8)		80 (79.2)	80 (79.2)	
≥110	2 (2.8)	3 (4.2)		21 (20.8)	21 (20.8)	
Lactate dehydrogenase (U/L)			1.000			1.000
< 245	69 (95.8)	68 (94.4)		60 (59.4)	60 (59.4)	
≥ 245	3 (4.2)	4 (5.6)		41 (40.6)	41 (40.6)	
C-reactive protein (g/mL)			0.314			0.362
<3.0	37 (51.4)	44 (61.1)		35 (34.7)	28 (27.7)	
≥3.0	35 (48.6)	28 (38.9)		66 (65.3)	73 (72.3)	
EBV-DNA (copies/mL)			0.860			1.000
<1000	49 (68.1)	47 (65.3)		10 (9.9)	10 (9.9)	
≥1000	23 (31.9)	25 (34.7)		91 (90.1)	91 (90.1)	

Abbreviations: ECOG PS, Eastern Cooperative Oncology Group Performance Status. CI, confidence interval; HR, hazard ratio; EBV, Epstein–Barr virus.

## Discussion

According to our knowledge, this is the first study to evaluate a prognostic nomogram based on EBV DNA, clinical features, hematology and biochemistry profiling as compared to clinical risk factors improves the ability to predict PFS in metastatic NPC patients. On the basis of the nomogram scores, we created a risk stratification system that stratified patients for PFS into low and high-risk groups. Moreover, the current prognostic model is a clinically useful tool for making personalized treatment recommendations for heterogeneous patient populations.

The National Comprehensive Cancer Network guidelines recommended platinum-combined chemotherapy is the primary treatment for recurrence and metastatic NPC (12). Recently, three large phase III trials have demonstrated that chemotherapy combined with PD-1 inhibition offered superior PFS for patients with recurrence and metastasis NPC compared to chemotherapy alone with a manageable safety profile (9, 10, 13). The results suggested that combined chemoimmunotherapy was a promising therapeutic strategy and would be a new standard of care for patients with recurrence and metastatic NPC. However, differences in clinical characteristics, the benefits of PD-1 inhibitor are not equally applied to all patients. Hence, there is an urgent to the identify patients with recurrence and metastatic NPC who are suitable for receiving combined PD-1 inhibitor.

Previous studies have shown that PD-L1 expression on tumor cells is favorably associated with anti-PD-1/PD-L1 therapy in a variety of malignancies (14, 15), but its predictive value in NPC is controversial. In the POLARIS-02 study, tumor response rates were similar (27.1 versus 19.4%) in NPC patients with PD-L1-positive and -negative for toripalimab monotherapy (16). The JUPITER-02 study showed that the clinical benefits of the toripalimab and chemotherapy combination were observed regardless of PD-L1 expression status (9). Additionally, EBV often present with intensive lymphocyte infiltration and overexpression of PD-L1. Xu et al. reported that plasma EBV DNA could be a useful biomarker for outcomes in patients with recurrence and metastatic NPC who are receiving anti-PD-1 therapy (17). The POLARIS-02 study showed that patients with pretreatment EBV DNA titers less than 10,000 IU/mL did not have a significantly higher clinical response than those with EBV DNA titers more than 10,000 IU/mL (16). In the CAPTAIN-1st study, longer survival was observed in patients with negative plasma EBV DNA compared with positive plasma EBV DNA in the camrelizumab and chemotherapy group (10). Currently, no prognostic models have tried to identify the ideal chemotherapy combination PD-1 inhibitor candidates.

As anatomies are insufficient in this study, we analyzed EBV DNA, clinical features, hematology and biochemistry profiling to explore the significance of a systemic environment in predicting patient outcome. In our study, baseline clinical characteristics such as the number of metastatic sites, liver metastases, and disease status were significantly related to survival, which were consistent with the results from previous reports (18, 19). Our previous work has demonstrated that rising baseline levels of alkaline phosphatase and lactate dehydrogenase in metastatic NPC (20, 21). Beyond pretreatment alkaline phosphatase and lactate dehydrogenase, unfavorable EBV DNA is also an adverse prognosticator for survival outcomes (18, 22). These findings were confirmed in the current study. To predict the risk of disease progression, a prognostic model was developed using these prognostic factors. In this study, the individualized risk stratified nomogram was used to identify subgroups where it would be advantageous for the addition of PD-1 inhibitor to chemotherapy to improve survival. Risk stratification based on the prognostic scores showed that PD-1 inhibitor plus chemotherapy significantly extended the median PFS for high-risk patients compared with chemotherapy alone. The finding was confirmed through the PSM method. Additionally, in spite of the balance in characteristics between the two arms, this benefit was not observed in low-risk patients. In the present study, high-risk group was more likely to present with higher baseline EBV DNA, liver metastases and multiple organ involvement. Interestingly, in the CAPTAIN-1st study, the subgroup analyses showed that a PFS benefit with PD-1 inhibitor was observed in patients with positive baseline EBV DNA, liver metastases and multiple organ (10). These characteristics might represent a high tumor burden state. In recent years, tumor mutational burden (TMB) was found to be correlated with clinical responses to anti-PD-1 therapy in most solid tumor (23, 24). A recent study by Zhang reported that NPC patients with high TMB displayed improved clinical responses to anti-PD-1 therapy, as measured by PFS (25). Therefore, these patients were more likely to be highly aggressive and more likely to benefit from immunotherapy. In contrast, the low-risk patient group might have been absence of liver metastasis, less metastatic organs, which provided evidence of a low tumor burden state. These patients were likely to be extremely sensitive to chemotherapy and present to a sustained state to chemotherapy. In this study, no significant difference was observed between chemotherapy combined PD-1 inhibitor and chemotherapy alone group in the patients with low-risk (P = 0.840). Whether low-risk patients need combined PD-1 inhibitor therapy still needs further exploration.

As far as we know, this is the first large comparative study examining the prognostic model among patients with metastatic NPC treated with immunotherapy and chemotherapy. However, some limitations remain in the work currently underway. This study is limited by the fact that since it is an observational study in real-life settings, potential selection bias is inevitable. However, this problem was minimized by recruiting all consecutively eligible patients and by using a large cohort of patients with metastatic NPC with a broad spectrum of baseline features adjusted for. Second, Due to the fact that the patients were enrolled from a single institution, there was no external validation of our model. It will strengthen our findings and generalize our model into clinical practice if it is externally validated. Third, the most common type of NPC in endemic areas is EBV-associated, and it may present different tumor features than those in low-risk areas. Considering these limitations, the validation of our proposed stratification model in other study settings is needed.

In conclusion, our novel prognostic model was able to stratify patients with metastatic NPC into low-risk or high-risk groups and identify candidates for combination PD-1 inhibitor therapy. Low-risk patients did not benefit from combination PD-1 inhibitor, but high-risk patients experienced significant survival benefits. These results are expected to be confirmed by a prospective clinical trial.

## Data availability statement

The original contributions presented in the study are included in the article/**Supplementary Material**. Further inquiries can be directed to the corresponding authors.

## Ethics statement

The study was approved by the Institutional Review Board of Sun Yat-Sen University Cancer Center. The patients/participants provided their written informed consent to participate in this study.

## Author contributions

Y-QX and H-RY designed the study. W-ZL and G-YL developed the methodology of study. W-XX, NL, W-XB, G-YL and HL participated in the acquisition of data. G-YL analyzed and interpreted the data. G-YL wrote the manuscript. All authors contributed to the article and approved the submitted version.
