# Supplementary material for: Development of a prognostic model to identify the metastatic nasopharyngeal carcinoma patients who may benefit from chemotherapy combination PD-1 inhibitor
Source: Front Immunol. 2023 Jan 17;14:1069010. doi: 10.3389/fimmu.2023.1069010 (PMC9887186; doi:10.3389/fimmu.2023.1069010)
Supplement: Supplementary file 1 [file DataSheet_1.docx]

**The supplementary data**

**Treatment:**

All eligible patients received one of the following platinum-based chemotherapy regimens as the first-line treatment: (1) PF: cisplatin (80-100 mg/m^2^ intravenously on Days 1 of a 21-day cycle) plus 5-fluorouracil (500 mg/m^2^ continuous intravenously infusion on Days 1–5 of a 21-day cycle); (2) TP: paclitaxel (175-200 mg/m^2^ intravenously on Day 1 of a 21-day cycle) or docetaxel (70-75 mg/m^2^ intravenously on Day 1 of a 21-day cycle) plus cisplatin (75-80 mg/m^2^ intravenously on Days 1 of a 21-day cycle); (3) TPF: paclitaxel (135 mg/m^2^ intravenously on Day 1 of a 21-day cycle) or docetaxel (60 mg/m^2^ intravenously on Day 1 of a 21-day cycle) plus cisplatin (60 mg/m^2^ intravenously on Days 1 of a 21-day cycle) plus 5- fluorouracil (300 mg/m^2^, continuous intravenously infusion on Days 1–5 of a 21-day cycle); (4) GP: Gemcitabine (75-80 mg/m^2^ intravenously on Day 1 of a 21-day cycle) plus cisplatin (80-100 mg/m^2^ intravenously on Days 1 of a 21-day cycle).

| **Supplementary Table 1. The baseline characteristics of high and low-risk groups in patients treated with chemotherapy plus PD-1 inhibitor or chemotherapy alone before propensity score matching.** | | | |
| --- | --- | --- | --- |
| Characteristic | High-risk group  (n=308, %) | Low-risk group (n=216, %) | *P* value |
| Gender |  |  | 0.120 |
| Male | 254 (82.5) | 166 (76.9) |  |
| Female | 54 (17.5) | 50 (23.1) |  |
| Age |  |  | 0.179 |
| <46 year | 124 (40.3) | 100 (46.3) |  |
| ≥46 year | 184 (59.7) | 116 (53.7) |  |
| ECOG PS |  |  | 1.000 |
| 0-1 | 291 (94.5) | 205 (94.9) |  |
| 2 | 17 (5.5) | 11 (5.1) |  |
| Smoking status |  |  | 0.114 |
| Yes | 117 (38.0) | 67 (31.0) |  |
| No | 191 (62.0) | 149 (69.0) |  |
| Type of metastasis |  |  | 0.534 |
| Primary metastatic | 138 (44.8) | 103 (47.7) |  |
| Recurrent metastatic | 170 (55.2) | 113 (52.3) |  |
| Number of metastatic sites |  |  | < 0.001 |
| Oligo | 5 (15.8) | 51 (23.6) |  |
| Multiple | 303 (84.2) | 165 (76.4) |  |
| Number of metastatic organs |  |  | < 0.001 |
| Single | 129 (41.9) | 170 (78.7) |  |
| Multiple | 179 (58.1) | 46 (21.3) |  |
| Liver metastatic |  |  | < 0.001 |
| Absent | 173 (56.2) | 177 (81.9) |  |
| Present | 135 (43.8) | 39 (18.1) |  |
| Lung metastatic |  |  | 0.174 |
| Absent | 178 (57.8) | 138 (63.9) |  |
| Present | 130 (42.2) | 78 (36.1) |  |
| Bone metastatic |  |  | 0.062 |
| Absent | 139 (45.1) | 116 (53.7) |  |
| Present | 169 (54.9) | 100 (46.3) |  |
| Lymph nodes metastatic |  |  | <0.001 |
| Absent | 206 (66.9) | 182 (84.3) |  |
| Present | 102 (33.1) | 34 (15.7) |  |
| Alkaline phosphatase (U/L) |  |  | < 0.001 |
| <110 | 237 (76.9) | 200 (92.6) |  |
| ≥110 | 71 (23.1) | 16 (7.4) |  |
| Lactate dehydrogenase (U/L) |  |  | <0.001 |
| < 245 | 189 (61.4) | 204 (94.4) |  |
| ≥ 245 | 119 (38.6) | 12 (5.6) |  |
| C-reactive protein (g/mL) |  |  | <0 .001 |
| <3.0 | 99 (32.1) | 130 (60.2) |  |
| ≥3.0 | 209 (67.9) | 86 (39.8) |  |
| EBV-DNA (copies/mL) |  |  | < 0.001 |
| <1000 | 25 (8.1) | 133 (61.6) |  |
| ≥1000 | 283 (91.9) | 83 (38.4) |  |

Abbreviations: ECOG PS, Eastern Cooperative Oncology Group Performance Status. CI, confidence interval; HR, hazard ratio; EBV, Epstein–Barr virus.
